# Diagnostic Accuracy of Diffusion-Weighted MRI for Differentiating Benign and Malignant Thyroid Nodules: Systematic Review and Meta-Analysis

**DOI:** 10.3390/cancers17162677

**Published:** 2025-08-18

**Authors:** Benjamin Noto, Carolin Bobe, Jonas Brandt, Heiner N. Raum, Nabila Gala Nacul, Burkhard Riemann, Anne Helfen

**Affiliations:** 1 Clinic for Radiology, University of Münster and University Hospital Münster, 48149 Münster, Germany; 2 Department of Nuclear Medicine, University of Münster and University Hospital Münster, 48149 Münster, Germany

**Keywords:** thyroid nodule, magnetic resonance imaging, diffusion magnetic resonance imaging, thyroid gland, meta-analysis

## Abstract

**Background**: Thyroid nodules are highly prevalent, affecting up to 75% of the population, yet most are benign. The limited specificity of ultrasound-based workup leads to substantial overdiagnosis and overtreatment, underscoring the need for improved imaging-based classification. Diffusion-weighted MRI (DWI), quantified via the apparent diffusion coefficient (ADC), has emerged as a promising imaging biomarker. This meta-analysis updates pooled diagnostic performance metrics and systematically evaluates which DWI acquisition techniques, imaging parameters, and combinations with other MRI modalities are most promising for clinical translation. **Methods**: PubMed, Web of Science, Scopus, and ProQuest were systematically searched. Pooled sensitivity, specificity, and area under the curve (AUC) were calculated using bivariate random-effects models. The effects of b-value, magnetic field strength, echo time, and diffusion model on diagnostic accuracy and ADC values were examined through subgroup and meta-regression analyses. **Results**: Forty-six studies (3003 nodules) were included. Pooled sensitivity and specificity were 0.84 (95% CI: 0.81–0.86) and 0.88 (95% CI: 0.85–0.90), with an AUC of 0.912. Intravoxel incoherent motion and diffusion kurtosis imaging showed no added value over the mono-exponential model. For the mono-exponential model, a negative association between b-values and reported ADCs was observed, whereas no association was found between b-values and diagnostic accuracy. Magnetic field strength and echo time did not affect ADCs. Combining DWI with morphological imaging showed the potential to further enhance diagnostic performance. **Conclusions**: DWI holds strong potential to improve the diagnostic workup of thyroid nodules. Technical standardization, particularly of key acquisition parameters, should be pursued to enable clinical implementation.

## Simple Summary

Thyroid nodules are highly prevalent, but most are benign. The limited specificity of current diagnostic approaches leads to unnecessary interventions and overtreatment. Diffusion-weighted MRI (DWI), which quantifies tissue microstructure using the apparent diffusion coefficient (ADC), is a promising non-invasive imaging modality for thyroid nodule classification. This meta-analysis of 46 studies demonstrates that DWI offers high diagnostic accuracy, with pooled sensitivity and specificity of 0.84 and 0.88, respectively. Evaluation of acquisition techniques and imaging parameters identified reduced field-of-view DWI and the mono-exponential ADC model as particularly promising for clinical application. However, the analysis also revealed a lack of technical standardization—especially regarding b-value selection—as a major hurdle to clinical translation. To fully realize the clinical potential of DWI, coordinated efforts toward standardizing acquisition protocols are needed.

## 1. Introduction

Thyroid nodules are a common clinical finding, with a prevalence of up to 75% in the general population, though only about 5–15% are malignant [1,2,3]. The primary aim of thyroid nodule workup is to accurately differentiate malignant from benign nodules. Ultrasound remains the first-line imaging modality in euthyroid patients, and several Thyroid Imaging Reporting and Data System (TIRADS) frameworks, based on B-mode ultrasound characteristics, have been developed to improve diagnostic consistency [4]. However, specificity remains a challenge: recent meta-analyses report specificities as low as 50% for the ATA Guidelines and around 70% for ACR-TIRADS [5]. Even the specificity of fine needle aspiration cytology (FNA) is limited. In a recent meta-analysis including 16,597 patients from 36 studies, FNA sensitivity was around 86%, but specificity was only 71% [6]. Given the high prevalence of benign nodules, this leads to substantial overdiagnosis and unnecessary interventions [7], underlining the need for improved diagnostic strategies.

Diffusion-weighted MRI (DWI) and its quantitative parameter, the apparent diffusion coefficient (ADC), offer a non-invasive means to probe tissue microstructure, with lower ADC values observed in malignant thyroid nodules due to higher cellular density and reduced extracellular space [8,9]. Thus, ADC may serve either as a standalone imaging biomarker or as a complementary measure integrated into existing frameworks like TIRADS.

Previous reviews and meta-analyses have demonstrated a high diagnostic performance of DWI for thyroid nodule classification [10,11,12]. However, these analyses have not accounted for the considerable methodological heterogeneity across studies, particularly in terms of technical parameters such as magnetic field strength and b-value selection. At least for the mono-exponential model, ADC values decrease with increasing b-values, making it essential to consider b-value dependence when comparing or applying thresholds. Moreover, previous reviews have not evaluated the diagnostic value of advanced diffusion models—such as intravoxel incoherent motion (IVIM) or diffusion kurtosis imaging (DKI)—or advanced acquisition techniques like reduced field-of-view (rFOV) DWI. This omission limits the interpretability and clinical translatability of the findings.

This meta-analysis builds on prior reviews by updating pooled diagnostic performance metrics while also introducing a critical additional dimension: a structured investigation into the impact of imaging parameters and techniques. By doing so, we seek to clarify the path toward standardizing DWI as a clinically useful and reproducible tool for thyroid nodule classification, and to identify a set of imaging parameters best suited for future research and clinical implementation.

## 2. Materials and Methods

The meta-analysis was conducted according to the Preferred Reporting Items for Systematic Reviews and Meta-Analyses of Diagnostic Test Accuracy (PRISMA-DTA) guidelines [13]. A systematic literature search was conducted in Pubmed, Web of Science, Scopus, and Proquest. No date limit was set. Search parameters used can be found in the Table A1. After removal of duplicates, a two-phase study selection process was used. First, titles and abstracts of retrieved articles were screened for relevance. Subsequently, the full texts of potentially eligible articles were reviewed in detail. This selection process was independently performed by two reviewers (A.H. and B.N.) on two separate occasions. Disagreements were resolved through consensus.

Study inclusion was based on the following PICOS criteria: Population (P): adults with thyroid nodules. Intervention (I): diffusion-weighted magnetic resonance imaging quantified by the apparent diffusion coefficient. Comparison (C): nodule dignity according to post-surgical histologic workup or fine needle aspirate cytology; Outcomes (O): sensitivity, specificity, mean ADC of benign and malignant nodules; Study Design (S): retrospective or prospective studies. Criteria for study-exclusion were as follows: (1) duplicate articles; (2) abstracts without full texts, editorial comments, letters, case reports, reviews, meta-analyses; (3) non-English full-text articles; (4) studies with incomplete or ambiguous data regarding sensitivity, specificity, or number of benign and malignant nodules; (5) studies with a highest b-value below 500 ${\mathrm{mm}}^{2}/s$, which falls short of the minimum highest b-value recommended by the Quantitative Imaging Biomarkers Alliance (QIBA) of the Radiological Society of North America for reliable ADC quantification [14].

Data was extracted by B.N. and independently cross-validated by J.B. and N.G.N. Discrepancies were resolved by consensus. The QUADAS-2 tool tailored to this review was applied by C.B. and independently cross-validated by B.N. to assess the quality of included studies and applicability concerns [15]. Disagreements were resolved by consensus discussion.

This review was not registered.

### Statistical Analysis

All analyses were conducted in R (version 4.1.0, R Foundation for Statistical Computing, Vienna, Austria). From the reported sensitivity, specificity, and number of malignant and benign cases, 2×2 contingency tables (true positive, false positive, true negative, false negative) were reconstructed.

Separate univariate random-effects meta-analyses for sensitivity and specificity were performed using the metaprop() function from the meta package [16], with logit transformation and inverse-variance pooling. Forest plots were generated to visualize study-level and summary estimates.

To jointly model sensitivity and specificity while accounting for their correlation, a bivariate random-effects meta-analysis via the reitsma() function from the mada package was applied [17]. Summary estimates and 95% confidence intervals were extracted, and an sROC curve was plotted, including individual study points and the pooled summary estimate with confidence region.

Subgroup analyses were conducted for studies using mono-exponential vs. IVIM-based DWI models.

To examine the effects of technical parameters (maximum b-value, magnetic field strength, echo time) on reported mean ADC values of studies using the mono-exponential ADC model, a weighted mixed-effects meta-regression was performed using the rma() function from the metafor package. The relative contribution of each predictor was quantified via changes in explained heterogeneity ($R^{2}$) when variables were removed from the full model.

## 3. Results

### 3.1. Literature Search

**Figure 1:**
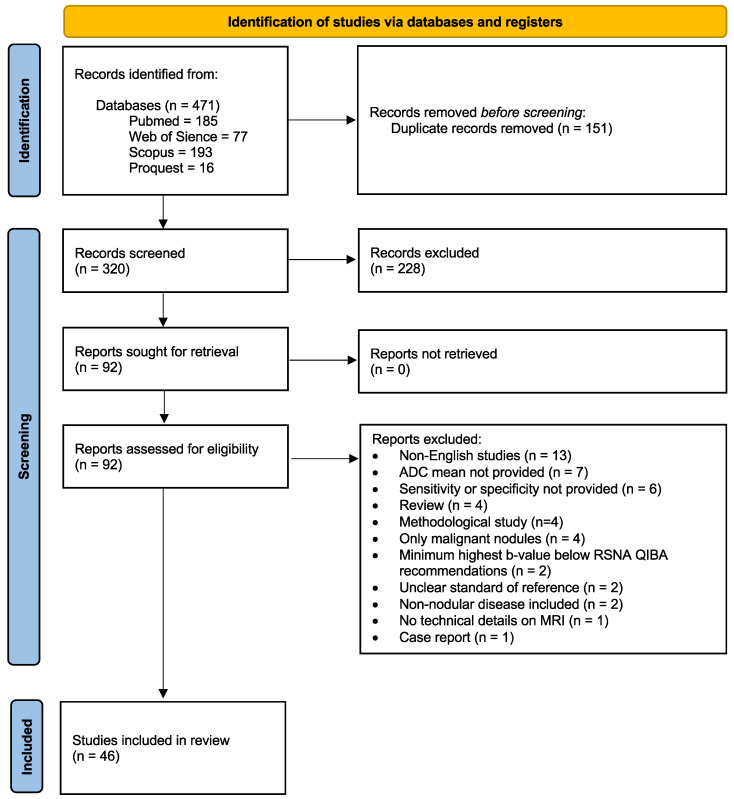
PRISMA flowchart showing the study selection process.

Figure 1 illustrates the flow of studies through the literature search and screening process in accordance with PRISMA guidelines and the predefined inclusion criteria.

### 3.2. Risk of Bias and Applicability Assessment

**Figure 2:**
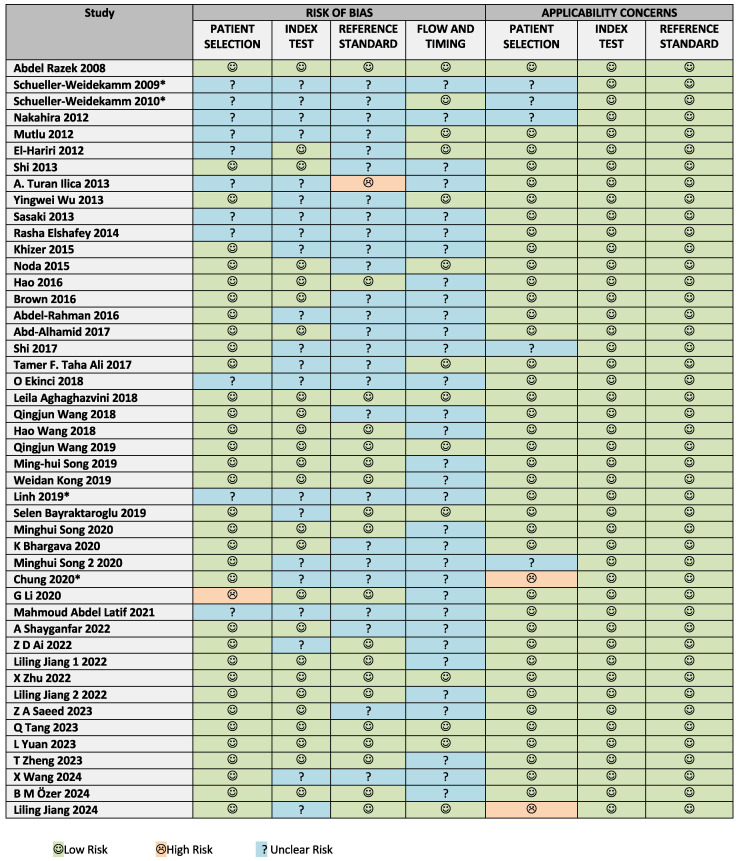
Figure summarizing the risk of bias and concern for applicability of the included 46 studies according to the QUADAS-2 tool, modified for this systematic review [8,9,18,19,20,21,22,23,24,25,26,27,28,29,30,31,32,33,34,35,36,37,38,39,40,41,42,43,44,45,46,47,48,49,50,51,52,53,54,55,56,57,58,59,60,61]. * Studies excluded from the analysis regarding the influence of the highest b-value, magnetic field strength, and echo time on reported ADC values because of reported higher ADC values for malignant nodules compared to benign nodules, contrary to the majority of the literature.

**Figure 3:**
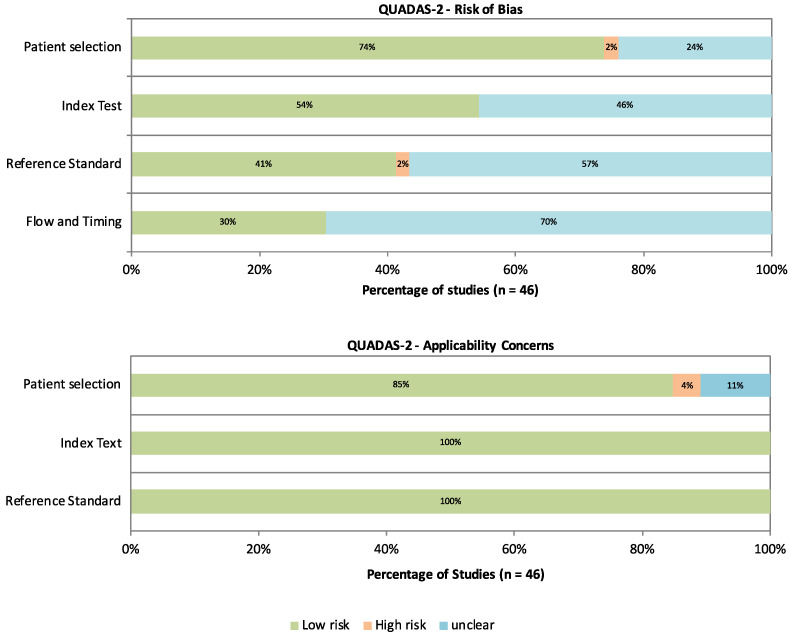
Graphical summary of the risk of bias and concern for applicability according to the modified QUADAS 2 tool.

**Figure A1:**
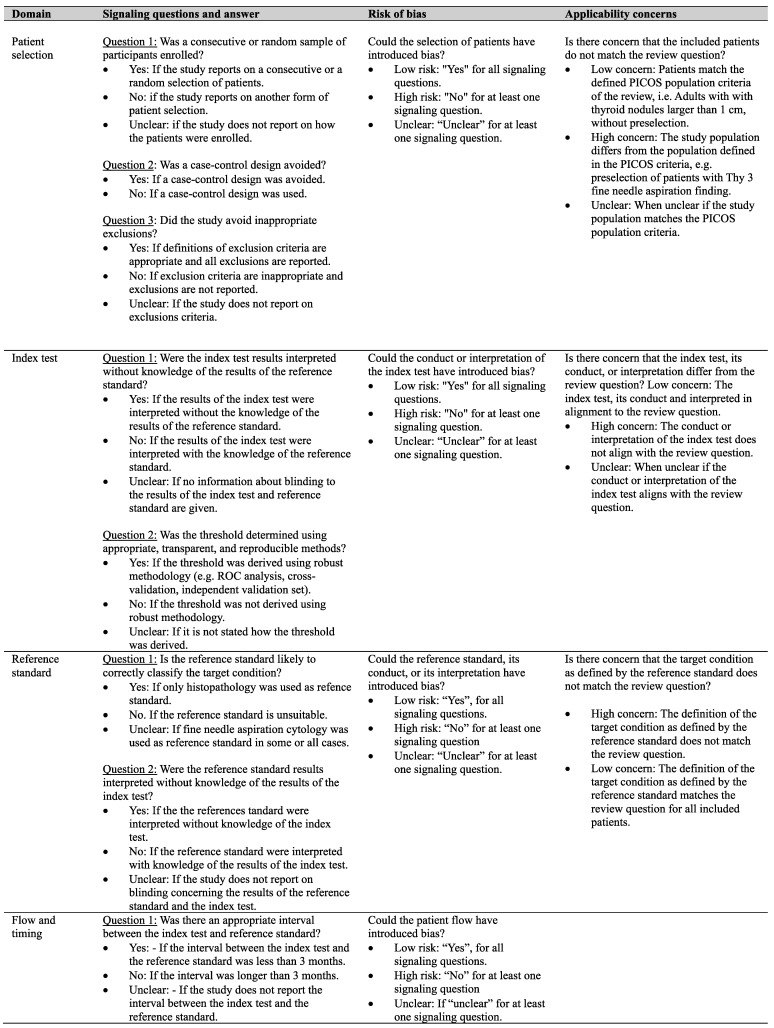
Items of the adapted QUADAS-2 tool and risk of bias and level of concerns about applicability.

Risk of bias and concerns regarding applicability were assessed using a modified version of the QUADAS-2 tool, as detailed in the appendix (Figure A1). The tool was adapted for this review by adding a tailored signaling question to the risk of bias domain: *Is the reference standard likely to correctly classify the target condition?* This was answered as follows: *Yes*, if only histopathology was used as the reference standard; *No*, if the reference standard was clearly unsuitable; and *Unclear*, if fine needle aspiration cytology (FNAC) was used as the reference standard in some or all cases. This modification reflects the limited diagnostic accuracy of FNAC compared to histopathology. Results are summarized in Figure 2 and visualized in Figure 3.

High risk of bias was rare. One study was rated as having a high risk of bias in the patient selection domain due to inappropriate exclusion criteria [18], and one study was deemed to have a high risk of bias in the reference standard domain due to the use of clinical follow-up as a reference in some cases [19]. Twelve out of forty-six studies (28.3%) used FNAC as the reference standard in some or all cases and were therefore rated as having unclear risk of bias in the reference standard domain. Apart from these specific concerns, most unclear ratings stemmed from insufficient reporting.

### 3.3. Study Characteristics

Forty-six studies with 3003 nodules (1746 benign and 1257 malignant) were included [8,9,18,19,20,21,22,23,24,25,26,27,28,29,30,31,32,33,34,35,36,37,38,39,40,41,42,43,44,45,46,47,48,49,50,51,52,53,54,55,56,57,58,59,60,61]. Concerning the standard of reference, 33 (71.7%) studies used histology as the sole reference, 8 (17.4%) used histology and FNA as references, 4 studies (8.7%) used FNA as the sole standard of reference, and one study (2.2%) used a combination of FNA and follow-up. Concerning magnetic field strength, 23 (50.0%) studies used MRI machines with a field strength of 1.5 Tesla, 21 (45.7%) studies with 3.0 Tesla, and two studies used a machine with 1.0 Tesla (4.3%). Countries of origin of the first authors were as follows: China (21), Egypt (7), Turkey (5), Japan (3), Pakistan (2), Austria (2), India (1), Iran (2), UK (1), Vietnam (1), and South Korea (1). Five studies applied IVIM [21,23,26,30,56]. For studies applying IVIM the diagnostic performance of *D* (pure diffusion) was assessed. For studies applying a mono-exponential diffusion model, the diagnostic performance of the ADC value based on the highest b-value was assessed.

### 3.4. Meta-Analysis of Diagnostic Performance

**Figure 4:**
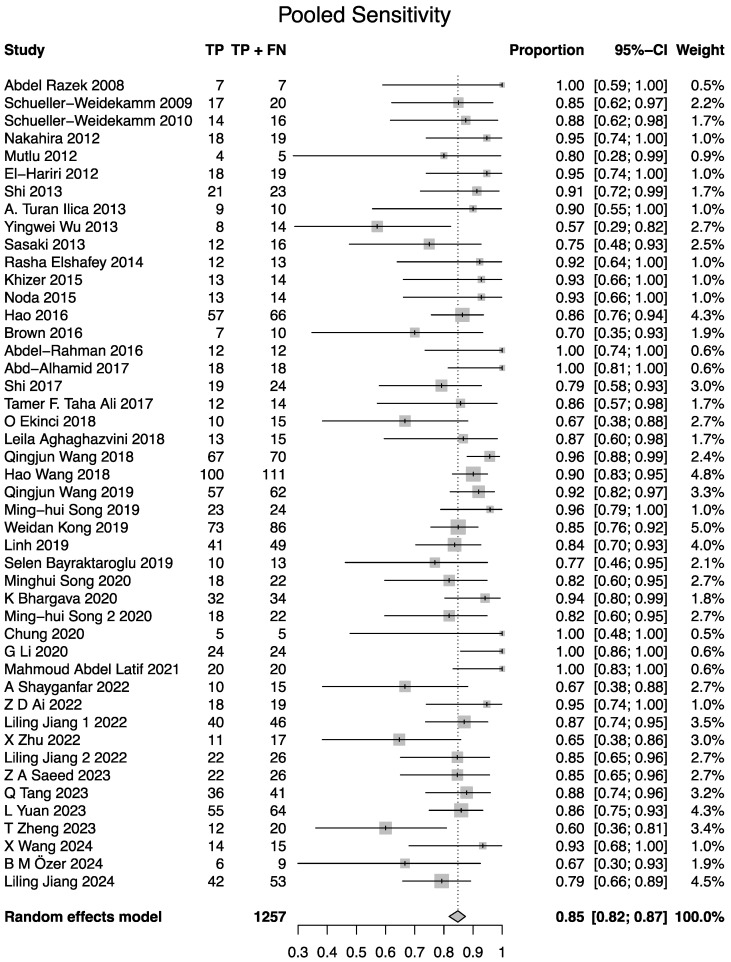
Forest plot for sensitivity (univariable analysis). Note the difference of 0.01 for the point estimate of pooled sensitivity in the univariable analysis compared to the bivariate analysis using the Reitsma method [8,9,18,19,20,21,22,23,24,25,26,27,28,29,30,31,32,33,34,35,36,37,38,39,40,41,42,43,44,45,46,47,48,49,50,51,52,53,54,55,56,57,58,59,60,61].

**Figure 5:**
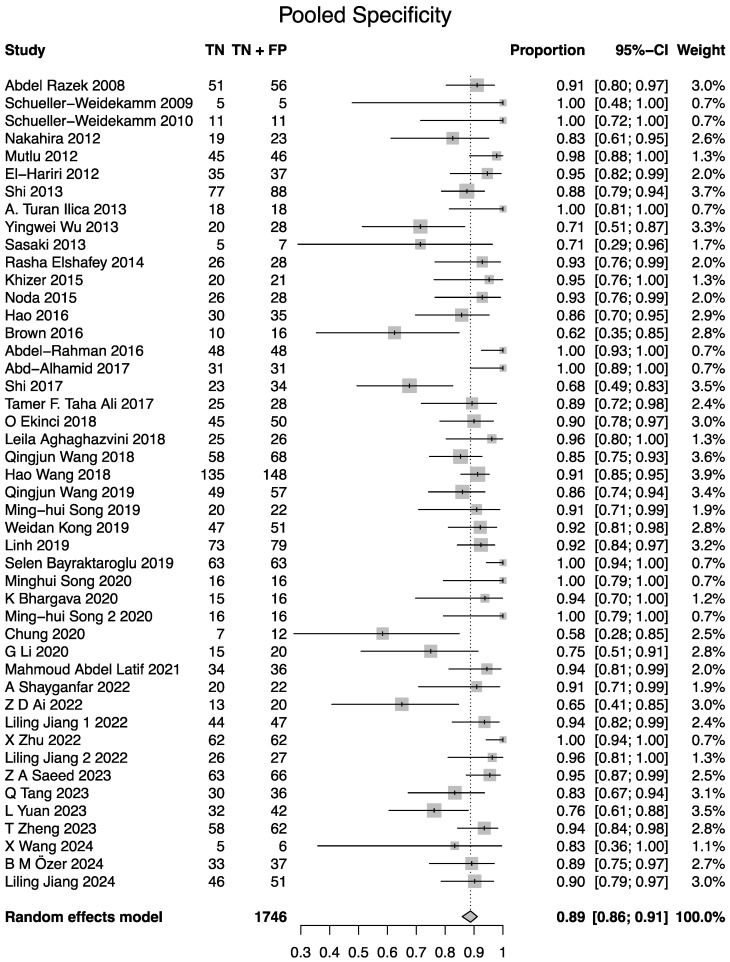
Forest plot for specificity. Note the difference of 0.01 in the univariable analysis of pooled specificity to the bivariate analysis conducted using the Reitsma method [8,9,18,19,20,21,22,23,24,25,26,27,28,29,30,31,32,33,34,35,36,37,38,39,40,41,42,43,44,45,46,47,48,49,50,51,52,53,54,55,56,57,58,59,60,61].

**Figure 6:**
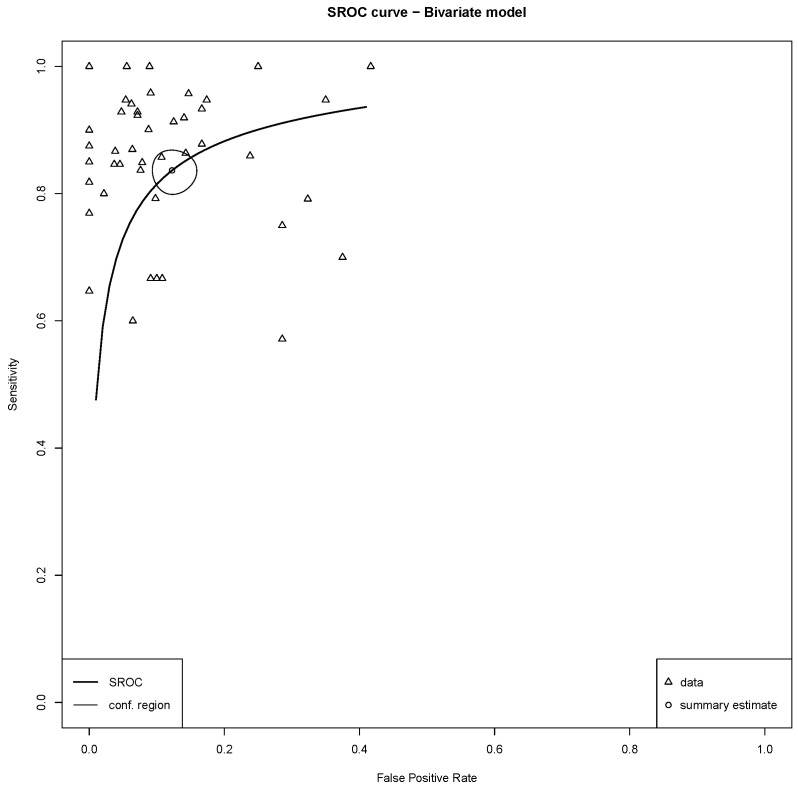
Summary receiver operating characteristic (sROC) curve from the bivariate meta-analysis of 46 included studies. The point estimates for the pooled sensitivity and specificity were 0.84 and 0.88, respectively. The area under the sROC curve was 0.91, indicating high overall diagnostic performance. The summary estimate and its 95% confidence region are shown.

All 46 studies demonstrated the diagnostic utility of DWI in differentiating benign from malignant lesions. The bivariate random-effects meta-analysis, using the Reitsma model, yielded a pooled sensitivity of 0.84 (95% CI: 0.81–0.86) and a pooled specificity of 0.88 (95% CI: 0.85–0.90). Forest plots and results of univariable analysis of sensitivity and specificity are presented in Figure 4 and Figure 5. The area under the summary receiver operating characteristic (sROC) curve was 0.91 (Figure 6). The normalized partial AUC, restricted to the observed false positive rates, was 0.85. Between-study heterogeneity, as quantified by $I^{2}$ statistics using the Zhou and Dendukuri approach, was 3.1% [62].

Subgroup analyses showed no statistically notable difference in the pooled sensitivity and specificity of studies using IVIM-based DWI compared to those using mono-exponential DWI (sensitivity: 0.87 vs. 0.84, p=0.537; specificity: 0.90 vs. 0.88, p=0.919).

Among studies using mono-exponential DWI, a bivariate meta-regression was conducted to assess whether the maximum b-value was associated with diagnostic performance. The maximum b-values reported in these studies ranged from 500 to 2000, with 1000 being most commonly used. No significant association was found between maximum b-value and either sensitivity (p=0.806) or specificity (p=0.397), indicating that variation in the b-value across studies did not meaningfully influence test accuracy.

### 3.5. Studies Reporting Lower ADC Values for Benign than for Malignant Nodules

In contrast to 42 studies reporting lower ADC values for malignant than for benign thyroid nodules, four studies—Schueller-Weidekamm et al. (2009 and 2010), Le Tuan Linh et al. (2019), and Chung et al. (2020)—were identified, that reported the opposite observation, namely lower average ADC values for benign nodules than for malignant ones [57,58,59,60]. Still, all four studies reported positively on the diagnostic capacity of DWI. A possible explanation for the findings of Chung et al. may lie in the specific composition of their study cohort, which purely included follicular neoplasms. In their analysis, follicular carcinomas demonstrated a higher mean ADC value ($0.783\times 10^{-3}\;{\mathrm{mm}}^{2}/s$ ) as compared to follicular adenomas ($0.581\times 10^{-3}\;{\mathrm{mm}}^{2}/s$) [60]. This is in contrast to findings reported by Abdel Razek and colleagues (2008), who observed a mean ADC of $0.77\times 10^{-3}\;{\mathrm{mm}}^{2}/s$ for follicular carcinoma, and a considerably higher value of $1.7\times 10^{-3}\;{\mathrm{mm}}^{2}/s$ for follicular adenoma [48]. The reasons for the reversed trend reported in the studies by the groups of Schueller-Weidekamm and Le Tuan Linh remain unclear.

### 3.6. Influence on Highest b-Value, Magnetic Field Strength, and Echo Time on Reported ADC Values

**Figure 7:**
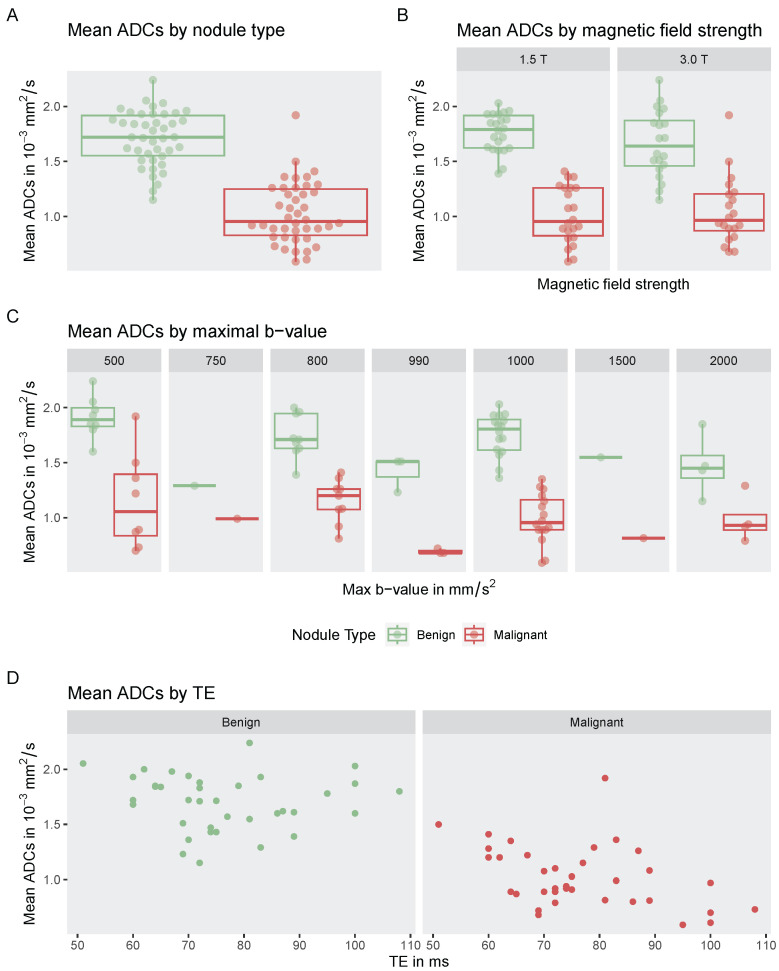
Mean ADC values (in $10^{-3}\;{\mathrm{mm}}^{2}/s$) reported by the included studies for (**A**) benign and malignant nodules, (**B**) stratified by magnetic field strength (in Tesla) of the MRI scanner used, and (**C**) stratified by the maximal b-value (in s/mm^2^) used in the respective studies. (**D**) Mean ADC values plotted against echo time (TE in ms), stratified by nodule type.

A weighted mixed-effects meta-regression was performed to examine the influence of imaging parameters (maximum b-value, magnetic field strength, echo time) and nodule type (benign vs. malignant) on reported mean ADC values. For this meta-regression, studies using IVIM (5 studies) were excluded. Also, the studies by Abdel-Rahman et al. (2016), Abd-Alhamid et al. (2016), Saeed et al. (2023), and Wang et al. (2024) were excluded since no standard deviations of mean ADC values were provided, which are necessary for meta-regression calculations [22,25,38,49]. Furthermore, the the studies by Schueller-Weidekamm et al. (2009 and 2010), Le Tuan Linh et al. (2019), and Chung et al. (2020), that reported lower ADC values for benign nodules as compared to to malignant ones, in contrast to all other studies, were excluded [57,58,59,60]. Hence, 33 studies were included. The impact of nodule type, b-value, magnetic field strength, and echo time (TE) on ADC values is visualized in Figure 7.

For the included studies the pooled estimate of the mean ADC was $1.76\times 10^{-3}\;{\mathrm{mm}}^{2}/s$ (95% CI: 1.68–1.85) for benign nodules and $1.08\times 10^{-3}\;{\mathrm{mm}}^{2}/s$ (95% CI: 0.98–1.18) for malignant. The meta-regression model explained 71.1% of the residual heterogeneity in reported ADC values ($R^{2}=0.71$). Higher maximum b-values were significantly associated with lower mean ADC values ($\beta =-0.0003\times 10^{-3}\;\frac{{\mathrm{mm}}^{4}}{s^{2}}$, p<0.001), while higher magnetic field strength and echo time showed no statistically notable association with ADC values ($\beta =0.0606\times 10^{-3}\;\frac{{\mathrm{mm}}^{2}}{s\times T}$, p=0.208) and ($\beta =-4.5620\times 10^{-3}\;\frac{{\mathrm{mm}}^{2}}{s^{2}}$, p<0.081). Malignant nodules had substantially lower mean ADC values compared to benign nodules ($\beta =-0.6801\times 10^{-3}\frac{\;{\mathrm{mm}}^{2}}{s}$, p<0.001). Analysis of relative variable importance showed that nodule type accounted for 89.2% of the explained variance, followed by maximum b-value (9.7%), magnetic field strength (0.6%) and echo time (0.6%).

### 3.7. Studies Reporting on Multimodal Analysis

Several studies evaluated the added diagnostic value of combining diffusion-weighted imaging (DWI) with other MRI-based techniques for thyroid nodule classification:

- **T1 Mapping:** Yuan et al. (2023) assessed the feasibility of combining T1 mapping with ADC measurements. They reported an AUC of 0.837 for ADC and 0.845 for T1 mapping, with a combined AUC of 0.956. Notably, the acquisition time for T1 mapping was only 36 s [32].
- **Morphologic Parameters:** Tang et al. (2023) integrated DWI metrics with morphological features commonly assessed in TIRADS. A combined model incorporating mean diffusivity from diffusion kurtosis imaging, maximum diameter, and margin irregularity achieved an AUC of 0.996, with a sensitivity of 95.1% and specificity of 100.0% [29].
  Wang et al. (2018) explored a multivariable model combining DWI with post-contrast and morphologic parameters. Independent predictors included ADC, irregular shape, a ring sign in the delayed phase, and cystic degeneration. An ADC-only model achieved an AUC of 0.95, while a combined model reached an AUC of 0.99 [45].
- **Amide Proton Transfer-Weighted Imaging (APT):** Li et al. (2020) examined APT imaging in combination with DWI (44 nodules; 22 malignant and 22 benign). APT alone yielded an AUC of 0.835. While ADC alone achieved an AUC of 0.95, the addition of APT did not further improve diagnostic performance (combined AUC: 0.95) [18].
- **Dynamic Contrast-Enhanced Imaging (DCE):** Song et al. (2020) evaluated the incremental value of DCE imaging alongside IVIM-derived diffusion parameters. Pharmacokinetic modeling of DCE parameters produced modest diagnostic performance (AUC = 0.668 for $K^{\mathrm{trans}}$, and 0.682 for $K_{\mathrm{ep}}$). In contrast, the IVIM-derived diffusion coefficient *D* alone achieved an AUC of 0.969. A combined model ($D+K^{\mathrm{trans}}+K_{\mathrm{ep}}$) improved the AUC to 0.991, although it was not significantly different from the AUC of *D* alone [21].
  Similarly, Sasaki et al. (2013) proposed a stepwise diagnostic approach combining ADC and DCE time intensity curves. While the combined model showed an accuracy of 91%, ADC alone achieved the same accuracy, suggesting limited added value of DCE [51].
- **Spectroscopy:** Three studies, all conducted in Egypt, evaluated the diagnostic performance of MR spectroscopy (MRS) in addition to DWI [46,50,52]. All reported improved sensitivity and specificity when combining ADC with MRS, although none described how the combined models were constructed, and none tested whether the improvements were statistically significant. Two of the studies originated from the same institution [50,52]. El-Hariri et al. (2012) reported sensitivities and specificities of 94% and 95% for DWI, 94.7% and 89.2% for MRS, and 96% and 100% for the combined approach, respectively [52]. Elshafey et al. (2014) reported 96% sensitivity and 85% specificity for DWI, 96% and 92% for MRS, and 100% and 93% for the combination [46]. Taha Ali (2017), in a cohort of 42 nodules (28 benign, 14 malignant), used MRS to assess the presence of a choline peak. Reported sensitivity and specificity were 100% and 89.3% for MRS, 85.7% and 89.2% for DWI, and 100% and 96% for the combined method [50].

### 3.8. Studies Investigating Advanced DWI Techniques

Liling Jiang et al. (2022) compared reduced field-of-view (rFOV) DWI with simultaneous multislice readout-segmentation of long variable echo-trains DWI (SMS-RESOLVE-DWI). rFOV-DWI demonstrated superior image sharpness, fewer artifacts, and overall better image quality compared to SMS-RESOLVE-DWI [27].

Xiuyu Wang et al. (2023) evaluated multiplexed sensitivity-encoding diffusion-weighted imaging (MUSE-DWI) against conventional DWI. MUSE-DWI provided improved image quality, clearer thyroid contour delineation, and greater lesion conspicuity. The reported acquisition times were similar: 3 min 40 s for MUSE-DWI and 3 min 27 s for conventional DWI [25].

### 3.9. Studies Investigating Advanced Diffusion Models

Xian Zhu et al. (2022) assessed the diagnostic performance of several advanced diffusion models, including bi-exponential, stretched exponential, and diffusion kurtosis imaging (DKI), in comparison to the conventional mono-exponential DWI model. No improvement in diagnostic accuracy was observed for any of the advanced models over the standard approach [33].

Similarly, two additional studies directly compared DKI with the mono-exponential model and reported no significant differences in diagnostic performance [9,29]. However, DKI required substantially longer acquisition times—6 min 52 s versus 1 min 52 s for conventional DWI [29].

IVIM has also been compared with the mono-exponential model in two studies, with no difference in diagnostic accuracy between the IVIM-derived true diffusion coefficient (*D*) and ADC values obtained from mono-exponential DWI [23,56].

Another study compared the diagnostic performance of IVIM and DKI-derived parameters. Again, no difference in diagnostic performance was found [26].

Five studies examined the IVIM-derived perfusion fraction (*f*) in benign versus malignant nodules, yielding inconsistent findings. Four studies reported significantly higher perfusion fraction values in benign nodules [23,26,30,56], while one study found higher values in malignant nodules [21].

#### Studies Investigating the Influence of b-Value Choice on Diagnostic Performance

Three studies investigated the influence of b-value selection on the diagnostic performance of ADCs calculated using mono-exponential DWI models [28,33,44]. ADC values were derived from both two-point models—specifically using the combinations (b0 and b800), (b0 and b1000), and (b0 and b2000)—as well as from more complex multi-point models incorporating up to 13 b-values: 0, 30, 50, 80, 100, 150, 200, 400, 600, 800, 1000, 1500, and 2000 s/mm^2^.

Both Xian Zhu et al. (2022) and Qingjun Wang et al. (2019) reported no significant differences in diagnostic performance between ADCs calculated from different b-value combinations. In contrast, in a previous study, Qingjun Wang et al. (2018) found significantly lower AUCs for ADC (b0–800) compared to ADC (b0–2000) and ADC( b0–800–2000), with p-values of 0.047 and 0.041, respectively [44].

With regard to signal intensity ratios (SIR), Liling Jiang et al. (2024) described differences in signal intensity between benign and malignant nodules at a b-value of 1500 s/mm^2^, which were visible to the naked eye [53]. Similarly, Qingjun Wang et al. (2019) reported high diagnostic accuracy for SIR measured at b = 2000 s/mm^2^ (SIR_b2000_), achieving an AUC of 0.975 for differentiating malignant from benign thyroid micronodules.

## 4. Discussion

This systematic review and meta-analysis aimed to evaluate the diagnostic performance of DWI in differentiating benign from malignant thyroid nodules and to examine the influence of technical imaging parameters. Across 46 included studies comprising 3003 nodules, DWI demonstrated a high diagnostic accuracy, with a pooled sensitivity of 0.84 (95% CI: 0.81–0.86), a pooled specificity of 0.88 (95% CI: 0.85–0.90), and an area under the summary receiver operating characteristic (sROC) curve of 0.91.

Three previous meta-analyses have investigated the diagnostic performance of DWI for thyroid nodule classification, all reporting high diagnostic accuracy [10,11,12]. Compared to our study—which includes 17 more studies than the largest of these earlier reviews—each of the prior analyses found similarly high pooled sensitivities, with confidence intervals ranging from 0.85 to 0.94. Our slightly lower sensitivity estimate may be explained by our use of a bivariate random-effects model that accounts for the correlation between sensitivity and specificity, offering a more rigorous statistical approach. Pooled specificities in the earlier meta-analyses ranged from 0.83 to 0.98 within their respective confidence intervals. Our pooled specificity of 0.88 (95% CI: 0.85–0.90) is consistent with these findings. Studies included in previous reviews but excluded from our review are listed in Table A2. Overall, our finding of a high diagnostic performance of DWI for thyroid nodule classification is consistent with earlier meta-analyses.

The $I^{2}$ statistic from our bivariate meta-analysis was 3.1%, indicating low between-study heterogeneity and a high degree of consistency in diagnostic performance across studies, despite differences in patient populations and technical parameters. This suggests robustness and potential generalizability of our findings.

### 4.1. Beyond Previous Meta-Analyses: Analysis of Technical Parameters

In contrast to earlier meta-analyses, our study not only updates pooled diagnostic performance estimates but also includes a detailed review and meta-regression analysis on the influence of technical imaging parameters. We further assessed studies that combined DWI with other MRI-based techniques.

Among studies applying the mono-exponential model to calculate ADC values, our meta-regression revealed a statistically notable negative association between the highest b-value and the reported ADC values. This relationship is consistent with theoretical expectations [63]. These findings emphasize the importance of standardizing DWI acquisition protocols to enhance inter-study comparability, support the development of robust ADC thresholds, and ultimately enable clinical translation. The diagnostic performance of the ADC was not influenced by b-value choice. Still, some studies reported that high b-values (e.g., ≥1500 s/mm^2^) assisted in visually detecting malignant nodules on trace DWI images. That said, the relevance of this advantage for potential future clinical implementations may be limited. In our opinion, patients referred for DWI due to thyroid nodules would almost certainly have already undergone thyroid ultrasound. The role of MRI in this context would likely not be for initial detection but rather for further characterization of nodules that appear suspicious on ultrasound. Given the drawbacks of high b-value imaging (i.e., need for advanced hardware, longer acquisition times since more signal averages are required), physical high b-value imaging does not seem essential. If facilitation of malignancy detection is desired, calculated high b-value DWI images could be an option.

Several studies also evaluated advanced diffusion models, primarily IVIM and DKI. However, none demonstrated superior diagnostic performance over the standard mono-exponential model. Given their extended acquisition times and hardware demands, these advanced techniques offer no clear diagnostic advantage. Our analysis also found no evidence that magnetic field strength affects reported ADC values, suggesting it plays a negligible role in ADC variability and can be deprioritized in future standardization efforts.

Regarding DWI technique, our review suggests that reduced field-of-view DWI is currently the most suitable option, as it provides improved image quality and lesion conspicuity, clinically acceptable acquisition times, and minimizes artifacts from surrounding anatomical structures.

In conclusion, our findings support the following DWI configuration as the most promising for future research and potential routine implementation: reduced field-of-view DWI using mono-exponential ADC calculation with a maximum b-value in the range of 500–1000 mm^2^/s.

### 4.2. Combination with Other Imaging Techniques

Several studies included in this review have investigated the feasibility of combining DWI with other MRI imaging techniques. In our opinion, the most obvious option is the combination with morphological image properties, following the established parameters of the ultrasound-based TIRADS systems. Two studies included in this review report near-perfect AUC for models combining morphological imaging parameters with DWI [29,45].

Thus, further combined research approaches on prediction models based on DWI-MRI and morphological parameters are highly desired.

### 4.3. Limitations

This study is limited by including only studies published in English, resulting in the exclusion of 13 potentially relevant studies. Also, some studies included in this meta-analysis were published prior to the recognition of the pathological entity known as noninvasive follicular thyroid neoplasm with papillary-like nuclear features (NIFTP). As a result, nodules that would now be classified as benign may have been previously categorized as malignant, potentially affecting the diagnostic accuracy estimates [64].

As previously discussed, the most probable clinical role of MRI lies in the further characterization of thyroid nodules that appear suspicious on ultrasound. However, only eight studies included in this review explicitly restricted inclusion to nodules indicated for FNA, either according to the American Thyroid Association Guidelines or a TIRADS classification system, limiting the applicability of this meta-analysis to the most likely real-world clinical use case [9,23,28,30,38,44,60,61]. Although all eight studies reported favorable diagnostic performance for DWI, further research specifically targeting nodules recommended for FNA under the current TIRADS appears warranted. Moreover, only one study focused exclusively on nodules classified as Bethesda category III or IV following FNA [60]. Notably, in many studies, prior needle biopsy was an explicit exclusion criterion [25,27,32,53]. Therefore, future investigations are needed to determine whether DWI remains feasible and diagnostically useful after needle biopsy, or whether post-bioptic changes diminish its value.

While our meta-analysis focused on diagnostic performance, it is important to acknowledge that resource availability, cost, and practicality of implementing MRI as a second-line diagnostic tool must also be considered. The feasibility of such use is likely to vary significantly across healthcare systems, depending on factors such as local infrastructure, reimbursement policies, and individual patient insurance coverage. These context-dependent factors should be taken into account when considering the broader clinical applicability of our findings.

### 4.4. Conclusions

In conclusion, the apparent diffusion coefficient represents a highly promising quantitative imaging biomarker for thyroid nodule classification. Provided that technical parameters can be standardized, DWI should seriously advance toward routine clinical application. Moreover, future research exploring the combined diagnostic value of DWI with morphological imaging features seems to hold considerable potential to further improve diagnostic accuracy.

## Data Availability

The data of this study are available from the corresponding author upon reasonable request.

## Abbreviations

The following abbreviations are used in this manuscript:

ACR	American College of Radiology
ADC	Apparent diffusion coefficient
ATA	American Thyroid Association
AUC	Area under the receiver operating curve
cDWI	Conventional single-shot spin echo echo-planar imaging
DCE	Dynamic contrast-enhanced MR perfusion
DKI	Diffusion kurtosis imaging
DWI	Diffusion-weighted MRI
EPI DWI	Echo-Planar Imaging DWI
FNA	Fine needle aspiration (cytology)
IVIM	Intravoxel incoherent motion
MRI	Magnetic resonance imaging
MRS	Magnetic Resonance Spectroscopy
MUSE-DWI	Multiplexed sensitivity-encoding diffusion-weighted imaging
NIFTP	Neoplasm with papillary-like nuclear features
PRISMA-DTA	Systematic Reviews and Meta-Analyses of Diagnostic Test Accuracy
QIBA	Quantitative Imaging Biomarkers Alliance
rFOV	Reduced field-of-view DWI
SIR	Signal intensity ratios
SMS-RESOLVE-DWI	Simultaneous Multi-Slice Readout Segmentation of Long Variable
	Echo-Trains DWI
sROC	Summary receiver operating characteristic
TE	Echo time
TIRADS	Thyroid Imaging Reporting and Data System

## Appendix A

**Table A1:** Search strategies for each database.

Database	Search Query
PubMed	((“Thyroid nodule”[Mesh]) OR (Thyroid[Title/Abstract])) AND (“Diffusion Magnetic Resonance Imaging”[Mesh] OR (diffusion-weighted[Title/Abstract]) OR (DWI[Title/Abstract]))
Web of Science	((TS=(DWI)) OR TS=(diffusion-weighted)) AND TS=(Thyroid nodule)
Scopus	TITLE-ABS-KEY (thyroid) AND TITLE-ABS-KEY (nodule) AND TITLE-ABS-KEY (diffusion) AND (LIMIT-TO (DOCTYPE, “ar”) OR LIMIT-TO (DOCTYPE, “re”) OR LIMIT-TO (DOCTYPE, “cp”))
ProQuest	noft(thyroid) AND noft(diffusion) AND noft(Magnetic resonance imaging) AND noft(nodule)

**Table A2:** Comparison with previous reviews.

	This Meta-Analysis	Lian-Ming Wu et al. (2014) [10]	Lihua Chen et al. (2016) [11]	Meyer et al. (2021) [12]
Number of included studies	46	7	15	24
Number of Nodules	3003	358	765	1714
Studies exluded from our review and reasons		[65]: Only available in Chinese. [66]: Could not be found based on the citation in the review.	[66]: Could not be found based on the citation in the review. [65,67,68]: Only available in Chinese.	[69]: Also included lymph nodes besides thyroid nodules. [70,71]: Insufficient maximum b-value. [71,72,73,74]: Reported only ADC values but not sensitivity and specificity. [75,76]: Included only malignant nodules. [77]: Unclear standard of reference.
Pooled sensitivity	0.84 (95 % CI: 0.81–0.86)	0.91 (95 % CI: 0.87–0.94)	0.90 (95 % CI: 0.85–0.93)	-
Pooled specificity	0.88 (95 % CI: 0.85–0.90)	0.93 (95 % CI: 0.86–0.96)	0.95 (95% CI: 0.88–0.98)	-
AUC	0.91	0.94	0.95	-
